# Hierarchical Gene Selection and Genetic Fuzzy System for Cancer Microarray Data Classification

**DOI:** 10.1371/journal.pone.0120364

**Published:** 2015-03-30

**Authors:** Thanh Nguyen, Abbas Khosravi, Douglas Creighton, Saeid Nahavandi

**Affiliations:** Centre for Intelligent Systems Research (CISR), Deakin University, Geelong Waurn Ponds Campus, Victoria, 3216, Australia; National Institute of Genomic Medicine, MEXICO

## Abstract

This paper introduces a novel approach to gene selection based on a substantial modification of analytic hierarchy process (AHP). The modified AHP systematically integrates outcomes of individual filter methods to select the most informative genes for microarray classification. Five individual ranking methods including t-test, entropy, receiver operating characteristic (ROC) curve, Wilcoxon and signal to noise ratio are employed to rank genes. These ranked genes are then considered as inputs for the modified AHP. Additionally, a method that uses fuzzy standard additive model (FSAM) for cancer classification based on genes selected by AHP is also proposed in this paper. Traditional FSAM learning is a hybrid process comprising unsupervised structure learning and supervised parameter tuning. Genetic algorithm (GA) is incorporated in-between unsupervised and supervised training to optimize the number of fuzzy rules. The integration of GA enables FSAM to deal with the high-dimensional-low-sample nature of microarray data and thus enhance the efficiency of the classification. Experiments are carried out on numerous microarray datasets. Results demonstrate the performance dominance of the AHP-based gene selection against the single ranking methods. Furthermore, the combination of AHP-FSAM shows a great accuracy in microarray data classification compared to various competing classifiers. The proposed approach therefore is useful for medical practitioners and clinicians as a decision support system that can be implemented in the real medical practice.

## Introduction

A large number of genes cannot be possibly analysed by traditional methods. DNA microarray is a technique that enables researchers to analyse the expression of many genes speedily. DNA microarray includes a process that labelled mRNA from a test tube is spread onto the microarray, which is made up of thousands of spots. Each DNA spot, which represents one gene, contains multiple identical strands of DNA. The labelled mRNA molecules move around the microarray to find and stick to their perfect matches. The quantity of mRNA bound to each spot on the array specifies the expression level of the various genes.

The DNA microarray technology therefore assists scientists to discover the genetic causes of anomalies arising in the functioning of the human body. A DNA microarray allows researchers to identify all the differences in gene expression between two different cell types, e.g. between normal (healthy) and diseased (cancer) cells, in a single experiment. Applications of DNA microarray data for the classification of disease based on different patterns of gene expression play a pivotal role in medical research. Classification of microarray data is necessary for real clinical practice, particularly in diagnosis of heart disease, infectious disease and the study of cancer. This task raises a huge challenge to researchers in statistics and also computational intelligence due to the high-dimensional-low-sample nature of the microarray data.

Wu et al. [1] employed a Laplace naive Bayes model for microarray data classification. The method takes group effects into account and is robust to outliers, which are commonly seen in gene expression data because of either chemical or electrical reasons. Chopra et al. [2] otherwise used gene pair combinations as inputs to the cancer classification algorithms rather than original gene expression profiles.

Basford et al. [3] considered both supervised and unsupervised classification for microarray data. The supervised classification is to identify clusters of tissues on the basis of the genes whereas unsupervised technique deals with the clustering of genes based on the tissues. Alternatively, a computational protocol for predicting gene markers in cancer tissues is used for analysing multiple cancer types in Xu et al. [4].

Yu et al. [5] proposed an undersampling method using the idea of ant colony optimization to classify imbalanced DNA microarray data. Giugno et al. [6] in another approach introduced a microarray data classification method using association rules. The authors suggested that the transcript expression intervals competently demonstrate discriminate subtypes in the same class.

Recently, Reboiro-Jato et al. [7] constructed a web-based interactive tool to assess the discriminative classification performance of custom hypothesis in the form of biologically related gene sets. The tool is able to provide valuation information for diagnostic analysis and clinical management decisions.

Although many methods have been proposed for microarray data classification, they can only provide nonintuitive classification results, which are not comprehensive and applicable to clinicians in the real practice. The behaviour of classification technique needs to be understood by human using tools like linguistic rules. Fortunately, this task can be accomplished by the means of fuzzy logic, which was introduced in 1960s. Application of fuzzy logic can provide clinicians with better understanding of the data and explanations on how diagnosed results are given. Furthermore, fuzzy logic offers good capability to handle noisy/missing data, which is a common problem in microarray data [8–10].

Inspired by the above circumstances, this paper proposes a method using fuzzy standard additive model (FSAM) for cancer microarray data classification. To enhance the efficiency of FSAM in dealing with high-dimensional-low-sample microarray data, genetic algorithm (GA) is incorporated in the FSAM learning process to optimize the FSAM rule structure.

Before performing genetic FSAM for microarray data classification, a subset of the most informative genes must be selected from thousands of genes. We propose herein a novel gene selection by modifying the traditional analytic hierarchy process (AHP) [11] that can then be quantitatively deployed to integrate outcomes of a number of individual gene ranking methods.

## Gene Selection Methods

Microarray data commonly collected with the number of genes (often in thousands) is much larger than the number of samples. Many standard techniques therefore find inappropriate or computationally infeasible in analysing such data. The fact is that not all of the thousands of genes are discriminative and needed for classification. Most genes are not relevant and do not affect the classification performance. Taking such genes into account enlarges the dimension of the problem, leads to computational burden, and presents unnecessary noise in the classification practice [9]. Thus it is crucial to select a small number of genes, called informative genes, which can suffice for good classification. However, the best subset of genes is often unknown [12].

Common gene selection approaches are filter and wrapper methods. Filter methods rank all features in terms of their goodness using the relation of each single gene with the class label based on a univariate scoring metric. The top ranked genes are chosen before classification techniques are deployed. In contrast, wrapper methods require the gene selection technique to combine with a classifier to evaluate classification performance of each gene subset. The optimal subset of genes is identified based on the ranking of performance derived from deploying the classifier on all found subsets. The filter procedure is unable to measure the relationship between genes whilst the wrapper approach requires a great computational expense [13].

### Brief literature review of gene selection methods

There have been a number of gene selection techniques in the literature for DNA microarray data classification. Liu at al. [14] introduced an ensemble gene selection method based on the conditional mutual information for cancer microarray classification. Multiple gene subsets serve to train classifiers and outputs are combined by a voting approach.

Likewise, Leung and Hung [15] initiated a multiple-filter-multiple-wrapper approach to gene selection to enhance the accuracy and robustness of the microarray data classification. Liu et al. [16] suggested another method, called ensemble gene selection by grouping, to derive multiple gene subsets. The method is based on virtue of information theory and approximate Markov blanket.

Bolón-Canedo et al. [17] in another approach investigated a gene selection method encompassing an ensemble of filters and classifiers. A voting approach was employed to combine the outputs of classifiers that help reduce the variability of selected features in different classification domains.

On the other hand, Bicego et al. [18] proposed a hybrid generative-discriminative approach using interpretable features extracted from topic models for expression microarray data classification. Orsenigo and Vercellis [19] examined nonlinear manifold learning techniques for dimensionality reduction for microarray data classification. Likewise, Ramakrishnan and Neelakanta [20] studied an information-theoretics inspired entropy co-occurrence approach for feature selection for classification of DNA microarray data.

Recently, Du et al. [21] suggested a forward gene selection algorithm to effectively select the most informative genes from microarray data. The algorithm combines the augmented data technique and L_2_-norm penalty to deal with the small samples’ problem and group selection ability respectively.

In this paper, to enhance the robustness and stability of microarray data classifiers, we introduce a novel gene selection method based on a modification of the AHP. The idea behind this approach is to assemble the elite genes from different ranking gene selection methods through a systematic hierarchy.

The next subsections scrutinize background of common filter gene selection methods, which are followed by our proposal.

Note that the following gene selection methods are accomplished by ranking genes via scoring metrics. They are statistic tests based on two data samples in the binary classification problem. The sample means are denoted as μ_1_ and μ_2_, whereas σ_1_ and σ_2_ are the sample standard deviations, and *n*_1_ and *n*_2_ are the sample sizes.

### Two-sample t-test

The two-sample t-test is a parametric hypothesis test that is applied to compare whether the average difference between two independent data samples is really significant. The test statistic is expressed by:

$$t=\left(\mu_{1}-\mu_{2}\right)/\sqrt{\frac{\sigma_{1}^{2}}{n_{1}}+\frac{\sigma_{2}^{2}}{n_{2}}}$$ (1)

In the application of t-test for gene selection, the test is performed on each gene by separating the expression levels based on the class variable. The absolute value of *t* is used to evaluate the significance among genes. The higher the absolute value, the more important is the gene.

### Entropy test

Relative entropy, also known as Kullback-Liebler distance or divergence is a test assuming classes are normally distributed. The entropy score for each gene is computed using the following expression:

$$e=\frac{1}{2}\left[\left(\frac{\sigma_{1}^{2}}{\sigma_{2}^{2}}+\frac{\sigma_{2}^{2}}{\sigma_{1}^{2}}-2\right)+\left(\frac{1}{\sigma_{1}^{2}}+\frac{1}{\sigma_{2}^{2}}\right){\left(\mu_{1}-\mu_{2}\right)}^{2}\right]$$ (2)

After the computation is accomplished for every gene, genes with the highest entropy scores will be selected to serve as inputs to the classification techniques.

### Receiver operating characteristic (ROC) curve

Denote the distribution functions of *X* in the two populations as *F*_1_(*x*) and *F*_2_(*x*) The tail functions are specified respectively *T*_i_(*x*) = 1-*F*_*i*_(*x*), *i* = 1,2. The *ROC* is given as follows:

$$ROC\left(t\right)=T_{1}\left(T_{2}^{-1}\left(t\right)\right),t\in \left(0,1\right)$$ (3)

and the area between the curve and the straight line (AUC) is computed by:

$$AUC=\int_{0}^{1}ROC\left(t\right)dt$$ (4)

The larger the *AUC*, the less is the overlap of the classes. For gene selection application, genes with the greatest *AUC* thus will be chosen.

### Wilcoxon method

The Wilcoxon rank sum test is equivalent to the Mann–Whitney U-test, which is a test for equality of population locations (medians). The null hypothesis is that two populations enclose identical distribution functions whereas the alternative hypothesis refers to the case two distributions differ regarding the medians. The normality assumption regarding the differences between the two samples is not required. That is why this test is used instead of the two sample t-test in many applications when the normality assumption is concerned.

The main steps of the Wilcoxon test [22] are summarized below:

1. Assemble all samples of the two populations and sort them in the ascending order.
2. The Wilcoxon statistic is calculated by the sum of all the ranks linked with the samples from the smaller group.
3. The hypothesis decision is made based on the p-value, which is found from the Wilcoxon rank sum distribution table.

In the applications of the Wilcoxon test for gene selection, the absolute values of the standardized Wilcoxon statistics are employed to rank genes.

### Signal to noise ratio (SNR)

SNR defines the relative class separation metric by:

$$SNR\left(f_{i},c\right)=\frac{\mu_{1}-\mu_{2}}{\sigma_{1}+\sigma_{2}}$$ (5)

where *c* is the class vector, *f*_*i*_ is the *i*th feature vector. By treating each gene as a feature, we transform the SNR for feature selection to gene selection problem for microarray data classification.

SNR implies that the distance between the means of two classes is a measure for separation. Furthermore, the small standard deviation favours the separation between classes. The distance between mean values is thus normalized by the standard deviation of the classes [23].

### A novel gene selection by modified AHP

Each of the above criteria can be employed to derive the ranking of genes and then to select greatest ranking genes for classification methods. The confidence of using a single criterion for selecting genes is not always achieved. Considering which criterion should be used is diffident. This question inspires an idea of taking into account the ranking of all criteria in evaluating genes. Through this way, elite genes of each criterion would be systematically assembled to form the most informative and stable gene subsets for classification. It is a difficult practice to combine ranking of all criteria because the ranges of statistics of criteria are different. The criterion generates a higher range of statistics would dominate those with a lower range. In order to avoid this problem, we utilize AHP in evaluating genes. The AHP deployment is commonly dealt with qualitative criteria where their evaluations are derived from experts. Nevertheless, experts’ knowledge is often limited particularly when the problem being solved is carried out on a wide number of criteria referring to various knowledge areas. This advocates the use of quantitative criteria in the AHP. The following presents a novel proposal vis-à-vis a ranking procedure to utilize quantitative criteria to the AHP for gene selection problem. The criteria used herein are the five test statistics i.e. t-test, entropy, ROC, Wilcoxon, SNR.

**Fig 1:**
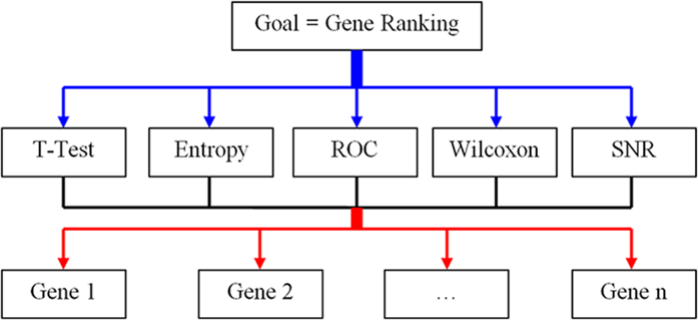
The hierarchy of factors for gene selection by AHP.

The AHP method as broadly applied in complex multi-criteria decision making is often performed with a tree structure of criteria and sub-criteria [24]. Due to the nature of the criteria selected here, the tree structure has three levels of hierarchies as illustrated in Fig. 1.

Five criteria are considered simultaneously during the AHP implementation. The five criteria are all quantitative so that we can intuitively put actual figures of these criteria into elements of the pairwise ranking matrix. This however would distort the matrix relative to other matrices describing assessments and judgements with respect to other criteria. Conventional applications of hierarchical analysis often draw on the Saaty rating scale [1, 9] and rough ratios, e.g. 1, 3, 5, 7, 9 to build pairwise comparison matrices [24, 25]. In this research, we propose the scale [1, 10] for ranking importance or significance of a gene compared with other genes. This scale will be applied to all criteria in the AHP application.

Suppose *X* = (*x*_*ij*_) is the *n*×*n*-dimension pairwise judgement matrix in which each element *x*_*ij*_ represents the relative importance of gene *i* over gene *j* with respect to a determined criterion, *n* is the number of genes. The reciprocal characteristic induces the following constraints

$$x_{ij}=1/x_{ji},\forall i\neq j,i,j\in \left[1,n\right]$$ (6)

$$x_{ii}=1,\forall i\in \left[1,n\right]$$ (7)

If gene *i* is absolutely more informative than gene *j*, then we have *x*_ij_ = 10. Accordingly gene *j* must be absolutely less important than gene *i* and *x*_ji_ = 1/10. Where *x*_ij_ = 1, this indicates that two genes are equally informative. The higher the value of *x*_ij_ϵ[1,10], the more important the gene *i* is in comparing with gene *j*. Element *x*_ij_ that is greater than 1 is called a superior element. Otherwise *x*_ij_ is called an inferior element as it is smaller than 1.

Let us define distance *d*_ij_ between two genes *i* and *j* with respect to a given criterion (e.g. t-test, entropy, ROC, Wilcoxon or SNR) by the absolute value of the subtraction between two statistics *c*_*i*_ and *c*_*j*_ of two genes.

$$d_{ij}=abs\left(c_{i}-c_{j}\right)\to d_{ij}=d_{ji}$$ (8)

Note that for all criteria, the higher the statistic, the more important the gene is. The procedure to acquire elements of comparison reciprocal matrices is described below where *c_max* is the maximum distance of genes regarding the given criterion, *c_max* = *max*(*d*_*ij*_),∀*i*,*j*∈[0,*n*], and *c* is a temporary variable.

#### Ranking procedure

FOR all pairs of two genes *i* and *j*

$$c=\frac{d_{ij}*9}{c\_max}+1=\frac{abs\left(c_{i}-c_{j}\right)*9}{c\_max}+1$$ (9)

IF (*c*_*i*_≥*c*_*j*_) THEN *x*_*ij*_ = *c* ELSE *x*_*ij*_ = 1/*c* END IF

END FOR

The expressions of x_ij_ ensure that superior elements of the judgment matrices will be distributed in the interval [1, 10]. Note that via calculations of the quantitative ranking method, the superior ratios are allowed to be real numbers within [1, 10] so that they can characterize more rigorously the judgement significance against the original Saaty rating scale. For example, consider four quantitative criteria A, B, C, and D with respective values 0.9, 1.3, 8.7, and 9.2. According to the Saaty rating scale, criteria B and A (D and C) are considered “equally important” and the ratios *x*_*BA*_ and *x*_*DC*_ will be equally assigned to 1: *x*_*BA*_ = *x*_*DC*_ = 1. Obviously, the difference between B and A (or D and C), though small, is neglected. However, with our ranking method, the ratios *x*_*BA*_ and *x*_*DC*_ are assigned more precisely and differently 1.4337 = *x*_*BA*_≠*x*_*DC*_ = 1.5422. Likewise, in the Saaty rating scale, criterion C is considered absolutely more important than criterion A and B, and the ratio *x*_*CA*_ and *x*_*CB*_ are both assigned 9. In our scale, the ratio *x*_*CA*_ and *x*_*CB*_ will be assigned differently 9.4578 and 9.0241 respectively. Hence the “absolute importance” judgement is relaxed and replaced by more rigorous judgements with different real numbers 9.4578 and 9.0241 rather than the same rough number 9 for both *x*_*CA*_ and *x*_*CB*._

After comparison matrices are constructed, hierarchical analysis calculates eigenvectors that demonstrate ranking scores of genes. Calculations of AHP are described succinctly in Table 1.

**Table 1:** AHP calculation procedure.

X	G_1_	…	G_n_	Sum of values	Eigenvector (*ϵ*)	Eigenvalue (*λ*)
G_1_	*x*_11_	…	*x*_1*n*_	*S*_1_ *=* (*x*_11_+ … +*x*_*n*1_)	$\epsilon_{1}=\left(\frac{x_{11}}{S_{1}}+\ldots +\frac{x_{1n}}{S_{n}}\right)/n$	$\lambda_{1}=\frac{\left[x_{11},\ldots ,x_{1n}\right].{\left[\epsilon_{1},\ldots ,\epsilon_{n}\right]}^{T}}{\epsilon_{1}}$
…	…	…	…	…	…	…
G_*n*_	*x*_*n*1_	…	*x*_*nn*_	*S*_*n*_ *=* (*x*_1*n*_+ … +*x*_*nn*_)	$\epsilon_{n}=\left(\frac{x_{n1}}{S_{1}}+\ldots +\frac{x_{nn}}{S_{n}}\right)/n$	$\lambda_{n}\;=\;\frac{[x_{n1},\ldots ,x_{nn}].{\left[\epsilon_{1},\ldots ,\epsilon_{n}\right]}^{T}}{\epsilon_{n}}$
					ϵ+ … +ϵ_*n*_ = 1	*λ*_*max*_ *=* max(*λ*_1_,…,*λ*_*n*_)

While applying the AHP, the matrix is required to be consistent and hence its elements must be transitive, that is *x*_*ik*_ = *x*_ij_ *x*_jk_. To verify the consistency of the comparison matrix X, Saaty [25] suggested calculating the Consistency Index (CI) and then Consistency Ratio (CR) based on large samples of matrices of purely random judgements. Let *ϵ* = [*ϵ*_1_,…, *ϵ*_*n*_]^T^ be an eigenvector and λ an eigenvalue of the square matrix *X*, so:

Xϵ=λϵ (10)

$$\text{Consistency Index }CI=\left(\lambda_{max}-n\right)/\left(n-1\right)$$ (11)

Consistency Ratio CR=CI/index (12)

CR should not exceed 0.1 if the set of judgements is consistent although CRs of more than 0.1 (but not too much more) sometimes have to be accepted in practice. CR equal to 0 implies the judgements are perfectly consistent.

When calculations for five criteria are completed, we obtain the so-called option performance matrix consisting of five eigenvectors that has the form shown in Table 2.

**Table 2:** Five eigenvectors of the option performance matrix.

	T-test	Entropy	ROC	Wilcoxon	SNR
Gene 1	ϵ_*T*1_	ϵ_*E*1_	ϵ_*R*1_	ϵ_*W*1_	ϵ_*S*1_
…	…	…	…	…	
Gene *n*	ϵ_*Tn*_	ϵ_*En*_	ϵ_*Rn*_	ϵ_*Wn*_	ϵ_*Sn*_

Finally the ranking of genes is the multiplication of the performance matrix and the vector representing the important weight of every criterion. The weight vector can be obtained by evaluating the important level of each criterion regarding the goal using the same procedure as described above. However, to avoid a bias judgement, we consider five criteria having an equally important level regarding the goal. Then the weight vector is (1/5; 1/5; 1/5; 1/5; 1/5)^T^. It is thus obvious that the ranking of genes is automatically normalized and it shows the important level of each gene taking into account not only a single criterion but all criteria simultaneously. Highest ranking genes are then selected for classification afterwards. In this paper, to testify the performance of classification techniques, a wide range number of genes is determined. Details of the number of genes selected are presented in the experimental section.

## Genetic Fuzzy System for Microarray Data Classification

### Fuzzy standard additive model (FSAM)

The FSAM system *F*: *R* ^*n*^ *→ R* ^*p*^ consists of *m* if-then fuzzy rules, which together can uniformly approximate continuous and bounded measurable functions in a compact domain [26, 27]. If-part fuzzy sets *A*_*j*_⊂*R* ^*n*^ can be any kind of membership functions. Likewise, then-part fuzzy sets *B*_*j*_⊂*R* ^*p*^ can be chose arbitrarily because FSAM utilizes only the centroid *c*_*j*_ and volume *V*_j_ of *B*_*j*_ to calculate the output *F*(*x*) given the input vector *x*ϵR^n^.

$$F\left(x\right)=Centroid\left(\sum_{j=1}^{m}w_{j}a_{j}\left(x\right)B_{j}\right)=\frac{\sum_{j=1}^{m}w_{j}a_{j}\left(x\right)V_{j}c_{j}}{\sum_{j=1}^{m}w_{j}a_{j}\left(x\right)V_{j}}=\sum_{j=1}^{m}p_{j}\left(x\right)c_{j}$$ (13)

Each of the *m* fuzzy rules in the word form *“If X = A*_*j*_ *Then Y = B*_*j*_ *”* is represented by a fuzzy rule patch of the form A_j_×B_j_⊂R^n^×R^p^. FSAM therefore graphically covers the graph of the approximand *f* with *m* fuzzy rule patches. If-part set *A*_*j*_⊂*R* ^*n*^ is characterized by the joint set function *a*_*j*_: *R* ^*n*^ *→*[0, 1] that factors: $a_{j}\left(x\right)\;=\;a_{j}^{1}\left(x_{1}\right)\ldots a_{j}^{n}\left(x_{n}\right)$. Then-part fuzzy set B_j_⊂R^p^ is similarly modelled by the membership function *b*_*j*_: *R* ^*p*^ *→* [0, 1] that has volume (or area) *V*_j_ and centroid *c*_*j*_. The convex weights expressed by:

$$p_{j}\left(x\right)=\frac{w_{j}a_{j}\left(x\right)V_{j}}{\sum_{k=1}^{m}w_{j}a_{k}\left(x\right)V_{k}}$$ (14)

induce the FSAM output *F*(*x*) as a convex sum of then-part set centroids. FSAM in particular or fuzzy system in general requires the order of *k* ^n+p-1^ rules to characterize the function *f*: *R* ^*n*^ *→ R* ^*p*^ in a compact domain.

Learning is a vital process of FSAM to construct a knowledge base that is a structure of if-then fuzzy rules. The FSAM learning process conventionally includes two basic steps: a) unsupervised learning for constructing if-then fuzzy rules and b) supervised learning for tuning rule parameters [28].

**Fig 2:**
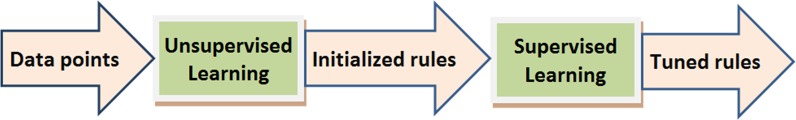
Hybrid system combines unsupervised and supervised learning.

The supervised learning often starts from a randomly initialized set of parameters and ends when it meets the determined stopping criteria. As training process costs much time and is often trapped in local minima, the initialization of parameters is thus a nontrivial issue. The unsupervised learning process, which is often accomplished by a clustering method, e.g. fuzzy c-means, helps to initialize parameters of fuzzy rules more skilfully (Fig. 2).

Microarray data normally associate with the high-dimensional nature that leads the FSAM classification to a rule explosion system facing the curse of dimensionality [29]. With a large number of rules, FSAM requires a large number of samples to train the system. This however contradicts with the low-sample characteristic of the gene expression microarray data. It is thus essential to optimize the rule structure to enhance efficiency of the learning process and the generalization capability of FSAM.

**Fig 3:**
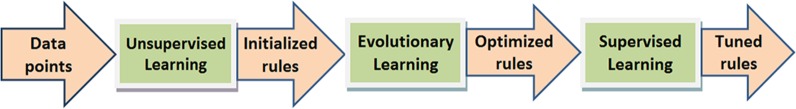
The evolutionary learning component in the learning process of FSAM.

In this paper, we propose the use of an evolutionary learning process, i.e. GA, to optimize the number of fuzzy rules before the supervised learning is performed. The evolutionary learning component is designed also to alleviate the computational cost of the succeeding supervised learning. The entire integration between GA and FSAM to formulate a genetic fuzzy system is illustrated in Fig. 3. Details of each learning component are presented in the following subsections.

### Unsupervised learning by the fuzzy c-means (FCM) clustering

The FCM clustering method [30] is applied to initialize parameters of FSAM. We organize the corresponding input and output data into a unique observation of p+1 dimensions where *p* is the number of inputs and one output corresponding to the class being classified. Denote *x*_i_ is the *i*th organized observation (*i* = 1,…,*N*), *x*_i_ is presented as follows:

$$x_{i}=[input_{i}^{1},input_{i}^{2},...,input_{i}^{p},output_{i}]$$ (15)

where ${input}_{i}^{j}$ is the *j*th input of the *i*th observation and *output_i_* is the output of the *i*th observation. By clustering the sample of *N* observations having the above format, we are able to derive the *C* resulting clusters corresponding with *C* fuzzy rules of the FSAM. Once the FCM clustering is completed, centres of the resulting clusters are assigned to centres of the membership functions (MFs). The centres of the output of each rule will be assigned equal to the output value of the corresponding cluster. The widths of the MFs of each rule are initialized based on the standard deviation of the data.

**Fig 4:**
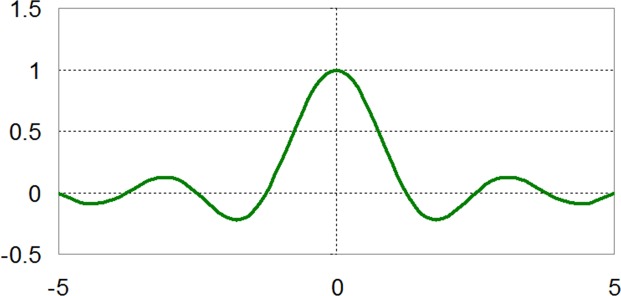
An example of the Sinc membership function.

The *sinc* membership function *sin*(*x*)/*x* recommended as the best shape for a fuzzy set in function approximation is used to construct if-then fuzzy rules [31]. The *j*th sinc set function (Fig. 4) centered at *m*_*j*_ and width d_*j*_ > 0 is defined as below:

$$a_{j}\left(x\right)=\sin\left(\frac{x-m_{j}}{d_{j}}\right)/\left(\frac{x-m_{j}}{d_{j}}\right)$$ (16)

Running the FCM clustering a number of times equal to the GA population size, we are able to obtain the initial population for GA, which is described in the following.

### Fuzzy rule structure optimization by GA

A GA [32] is an unorthodox search or optimization technique operated on a population of *n* artificial individuals. Individuals are characterized by chromosomes (or genomes) *S*_k_, *k = {*1,*…*,*n}*. The chromosome is a string of symbols, which are called genes, S_k_ = (S_*k1*_,…,S_*kM*_), and *M* is a string length. Individuals are evaluated via calculation of a fitness function. To evolve through successive generations, GA performs three basic genetic operators: selection, crossover and mutation.

A roulette wheel selection method is used to select the individuals that go on to produce an intermediate population. Parents are selected based on their fitness. Chromosomes have more chances to be selected if they are better (have higher fitness) than the others. Imagine all chromosomes in the population are placed on a roulette wheel, and each has its place big according to its fitness function.

The wheel is rotated and the selection point indicates which chromosome is selected when the wheel is stopped. It is obvious that the chromosome with bigger fitness will be selected more times (competing rule in the evolutionary theory).

The crossover operator selects random pairs from the intermediate population and performs 1-point crossover. Genes from parent chromosomes are selected to create new offspring.

Finally, individuals are mutated and they form the new population. The mutation prevents falling all solutions in the population into a local optimum of the problem being solved. A few randomly chosen bits are switched from 1 to 0 or from 0 to 1.

Through chromosomes’ evolution, GA searches for the best solution(s) in the sense of the given fitness function. We employ GA to train the complicated FSAM comprising many parameters. The fitness function is designed with the aim to reduce the number of fuzzy rules and also to decrease the learning error at the same time. The following formula is proposed:

$$fit(m)=ln({\bar{\sigma }}^{2})+\frac{log_{n}\left(m\right)}{n}$$ (17)

Where *m* is the number of fuzzy rules, *n* is the number of data samples, and ${\overset{-}{\sigma }}^{2}$ is the error term defined by the following equation:

$${\bar{\sigma }}^{2}=\frac{1}{n}\;\sum_{i\;=\;1}^{n}{\left(y_{i}-F\left(x_{i}\right)\right)}^{2}$$ (18)

where *y*_*i*_ is the real value and *F*(*x*_*i*_) is the output of the FSAM. Parameters of FSAM are coded into genes of the GA chromosomes/individuals. With a population of individuals, GA can simultaneously explore different parts of the training model’s parameter space and thus it is able to find the global solution to simultaneously minimize the error term and reduce the number of fuzzy rules.

### FSAM supervised learning

A supervised learning process is carried out to tune parameters of the FSAM system. The gradient descent algorithm can adjust all parameters in the FSAM. We attempt to minimize the squared error:

$$E\left(x\right)=\frac{1}{2}{\left[f\left(x\right)-F\left(x\right)\right]}^{2}$$ (19)

The vector function *f*: *R* ^*n*^ → *R* ^*p*^ has components *f*(*x*) [*f*_1_(*x*),…,*f*_*p*_ (*x*)] ^*T*^ and so does the vector function *F* Let $\xi_{j}^{k}$ represent the *k* ^*th*^ parameter in the membership function *a*_j_. Then the chain rule allows the gradient of the error function (Equation 19)with respect to $\xi_{j}^{k}$, with respect to the then-part set centroid$c_{j}={\left(c_{j}^{1},\ldots ,c_{j}^{p}\right)}^{T}$, and with respect to the then-part set volume *V*_*j*_ to be derived [28, 29].

A gradient descent learning rule for a FSAM parameter has the form:

$$\xi \left(t+1\right)=\xi \left(t\right)-\mu_{t}\frac{\partial E}{\partial \xi }$$ (20)

Where μ_*t*_ is the learning rate at iteration *t*.

Generally, there are two ways to adjust parameters: batch form refers to the update process that occurred when all training samples have completely passed through the system. Incremental form refers to the update that occurred as soon as a sample was processed. With significantly nonlinear data, incremental adjustment often proves effective and more stable, and it is therefore applied in this study.

The momentum technique is also integrated so as to enhance the convergent speed of the parameter tuning process [33]. The learning formula with momentum is given by:

$$\xi \left(t+1\right)=\xi \left(t\right)-\mu_{t}\frac{\partial E}{\partial \xi }+\varepsilon .\Delta \xi \left(t\right)$$ (21)

where ε is the momentum coefficent.

## Experimental Results

### Performance evaluation metrics

It is well-known that there are often a small number of samples in the gene expression microarray datasets. In order to train as many examples as possible, the leave one out cross validation (LOOCV) [34, 35] is organized. The strategy divides all samples at random into *K* separate subsets, where *K* is the number of samples. As with the traditional *k*-fold cross validation, this strategy uses *K*-1 subsets for training whilst the *k*-th sample is for testing. The LOOCV accuracy is computed as follows:

$$LOOCVaccuracy=\frac{ACC}{K}$$ (22)

where *ACC* is the number of correctly classified examples in *K* experiments.

Sensitivity and specificity are also employed to measure performance of classification techniques. The sensitivity of a test refers to the proportion of patients with disease who test positive. Conversely, specificity measures the proportion of patients without disease who test negative. Another important performance metric in medical application, which is area under the ROC curve (AUC) is also calculated.

### Detailed experiments

Two benchmark datasets used for experiments in this section are the diffuse large B-cell lymphomas (DLBCL) dataset [36] and the leukemia cancer dataset [23].

DLBCL and follicular lymphomas (FL) are two malignancies to be classified. The classification models are constructed using gene expression profiles to distinguish between these two lymphomas. The DLBCL dataset composes of 7070 genes and 77 samples where DLBCL contributes 75.3% with 58 examples and the rest 24.7% are of FLs with 19 samples.

The leukemia dataset is one of the most renowned gene expression cancer datasets. The dataset includes information on gene-expression in samples from human acute myeloid (AML) and acute lymphoblastic (ALL). The dataset consists of 5147 genes with 72 samples. ALL samples occupy 65.3% (47 samples) whereas AML contribute 34.7% (25 samples).

Six gene selection methods, i.e. t-test, entropy, ROC, Wilcoxon, SNR and our proposed modified AHP presented in section 2, are carried out to select most discriminative genes for classification. For the sake of comparisons with the FSAM, three other classification approaches including multilayer perceptron (MLP), support vector machine (SVM), and fuzzy ARTMAP (FARTMAP) are also deployed. Each of the classification techniques is performed on various numbers of the most significantly informative genes. The wide range number of genes selected includes [3; 6; 10; 15; 20; 30]. Too small or too large number of genes beyond the mentioned range would lead to a performance reduction.

In the data pre-processing step, some filter approaches are employed to remove genes with low absolute values, little variation, small profile ranges or low entropy. These genes are generally not of interest because their quality is often bad due to large quantization errors or simply poor spot hybridization [37]. The gene profiles are then normalized using the quantile normalization technique [38].

Different gene selection approaches result in different subsets of informative genes. The lists of 30 genes ranked top by each gene selection method in the DLBCL and leukemia datasets are assembled in S1 Table and S2 Table in the Supporting Information section. Table 3 and 4 show the overlap among 30 selected genes of six gene selection methods t-test, entropy, ROC, Wilcoxon, SNR and AHP.

**Table 3:** Overlap matrix among gene selection methods: the DLBCL dataset.

	T-test	Entropy	ROC	Wilcoxon	SNR	AHP
**T-test**	30	16	27	26	7	24
**Entropy**	16	30	14	14	2	18
**ROC**	27	14	30	**29**	10	**25**
**Wilcoxon**	26	14	29	30	11	**25**
**SNR**	7	2	10	11	30	12
**AHP**	24	18	25	25	12	30

**Table 4:** Overlap matrix among gene selection methods: the Leukemia dataset.

	T-test	Entropy	ROC	Wilcoxon	SNR	AHP
**T-test**	30	25	23	25	9	23
**Entropy**	25	30	24	25	11	24
**ROC**	23	24	30	**28**	15	**25**
**Wilcoxon**	25	25	28	30	13	**26**
**SNR**	9	11	15	13	30	16
**AHP**	23	24	25	26	16	30

It is seen that there is a great overlap between AHP and ROC or between AHP and Wilcoxon methods. In the DLBCL dataset, there are 25 common genes out of 30 selected genes between AHP and Wilcoxon and also between AHP and ROC (Table 3). Likewise, in the leukemia dataset, 25 common genes are found between AHP and ROC. This number is 26 between AHP and Wilcoxon (Table 4).

Notably, Wilcoxon and ROC share most of the genes selected in both datasets. The number of common genes between two methods is 29 and 28 out of 30 in the DLBCL and leukemia datasets respectively. The similarity of these two methods as well as their disconnection with t-test, entropy, and SNR are explainable as ROC and Wilcoxon are nonparametric tests that are not based on the normality assumption. On the other hand, SNR is the most dissimilar approach among the six investigated methods.

**Fig 5:**
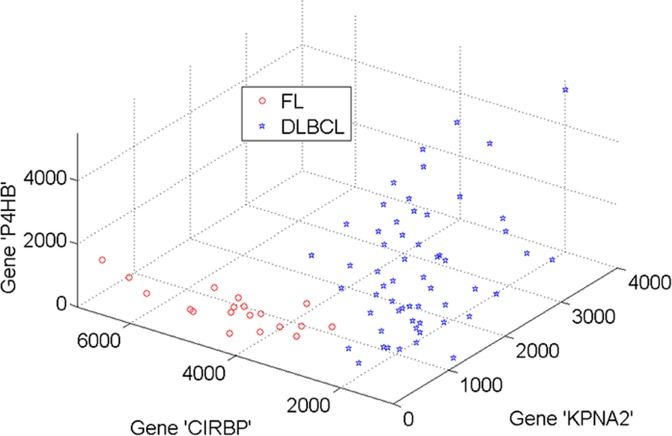
3D projection of three genes in the DLBCL dataset selected by AHP method.

**Fig 6:**
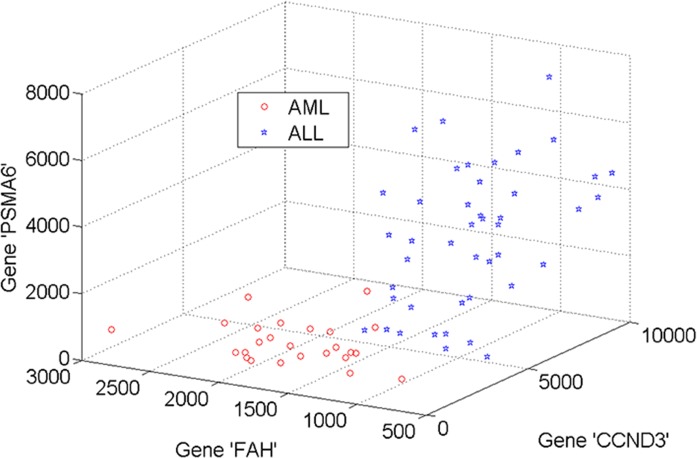
Three informative genes in the leukemia dataset selected by AHP method.

Fig. 5 and 6 demonstrate the 3D projections of three most informative genes selected by the modified AHP method in the DLBCL and leukemia datasets respectively. Subsets of genes selected by the AHP exhibit a clear separation between two classes. The selection of these subsets largely affects the performance of classification techniques deployed afterwards. The AHP selection of 3 genes is different from those of the other methods (see S1 Table and S2 Table). Rather than using outcomes of individual methods, AHP combines top informative genes across methods. For example, in the DLBCL dataset, three genes selected by AHP are ‘KPNA2’, “CIRBP’ and ‘P4HB’. Likewise, genes with symbols ‘CCND3’, ‘FAH’ and ‘PSMA6’ are selected in the leukemia dataset.

The average LOOCV accuracy across different numbers of genes of the four classification methods, i.e. MLP, SVM, FARTMAP and FSAM is reported in Table 5 and 6 for the DLBCL and leukemia datasets respectively. The statistics in brackets show the maximum performance associated with the corresponding number of genes right after the hyphen.

**Table 5:** LOOCV accuracy on the DLBCL dataset.

	T-test	Entropy	ROC	Wilcoxon	SNR	AHP
MLP	94.44 (98.61–30)	94.44 (98.61–30)	95.83 (98.61–6)	95.37 (97.22–10)	92.59 (95.83–20)	96.97 (98.70–3)
SVM	95.24 (97.40–6)	91.77 (97.40–30)	97.84 (100–10)	97.62 (100–3)	97.84 (98.70–3)	98.05 (100–3)
FARTMAP	93.51 (98.70–15)	94.37 (98.70–10)	92.86 (98.70–6)	94.81 (100–6)	96.10 (100–3)	97.19 (100–3)
FSAM	94.59 (97.40–10)	94.37 (98.70–15)	98.27 (100–6)	96.32 (98.70–10)	97.84 (100–10)	98.70 (100–6)

**Table 6:** LOOCV accuracy on the leukemia dataset.

	T-test	Entropy	ROC	Wilcoxon	SNR	AHP
MLP	95.83 (98.61–15)	95.14 (98.61–15)	96.30 (98.61–20)	95.14 (100–30)	91.20 (94.44–30)	97.69 (100–6)
SVM	93.75 (98.61–15)	94.68 (100–15)	93.29 (100–10)	94.91 (100–10)	88.19 (98.61–10)	96.06 (100–6)
FARTMAP	96.30 (98.61–3)	94.44 (98.61–30)	96.53 (98.61–6)	96.30 (98.61–6)	94.44 (98.61–15)	96.76 (100–6)
FSAM	96.99 (100–10)	96.53 (98.61–30)	97.22 (100–10)	97.92 (100–15)	95.60 (100–15)	98.38 (100–15)

## Discussions

It is found that there is a great dominance of the AHP gene selection method compared to the other investigated gene selection methods. This is recognized by averaging the LOOCV accuracy across four classification methods for each gene selection method.

**Fig 7:**
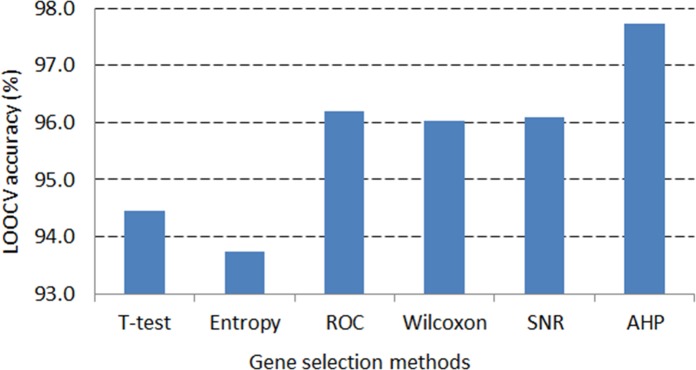
LOOCV accuracy average across classifiers in the DLBCL dataset.

**Fig 8:**
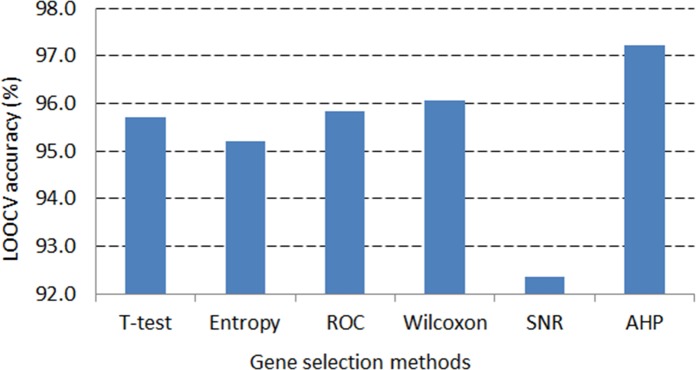
LOOCV accuracy average across classifiers in the Leukemia dataset.

In the DLBCL dataset, the application of AHP method on average generates nearly 98% LOOCV accuracy that is the greatest statistic compared to those of the remaining gene selection methods (see Fig. 7). Likewise, in the leukemia dataset, the highest LOOCV accuracy, more than 97%, is also resulted from the AHP gene selection method (see Fig. 8). This demonstrates the robustness of the AHP gene selection method as it can produce high classification performance regardless of classification techniques or number of genes selected.

ROC and Wilcoxon are the second best methods, which can result in high classification accuracy just after the AHP approach. The overlap of selected genes among the ROC, Wilcoxon and AHP criteria explains the performance resemblance among them (see Table 3 and 4).

On the other aspect, it is seen that FSAM demonstrates the greatest average LOOCV accuracy across gene selection criteria among investigated classification methods.

**Fig 9:**
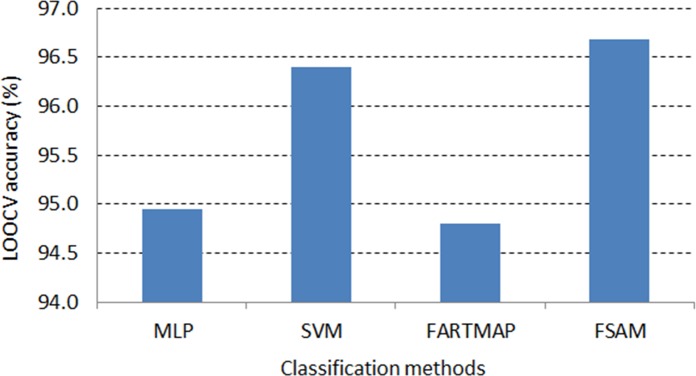
Average LOOCV accuracy across gene selection methods in DLBCL dataset.

**Fig 10:**
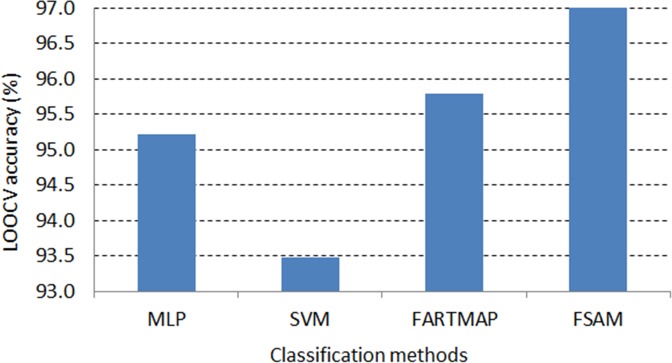
Average LOOCV accuracy across gene selection methods in leukemia dataset.

Fig. 9 and 10 exhibit the dominance of FSAM against the other methods. In both DLBCL and leukemia datasets, FSAM achieves approximately 97% accuracy on average across gene selection methods. SVM performs rather competently in the DLBCL dataset but it is the worst in the leukemia dataset. MLP and FARTMAP show approximate performance as MLP dominates FARTMAP in the DLBCL dataset whilst the reverse performance is found in the leukemia dataset.

Notably, FSAM can classify correctly all testing samples with absolutely 100% accuracy with some gene selection methods. For example, in the DLBCL dataset, 6 top ranked genes selected by the ROC or AHP can lead to maximum accuracy by the FSAM classifier. Likewise, 10 genes selected by the SNR method can also serve FSAM to obtain 100% accuracy (see Table 5). Alternatively, in the leukemia dataset, 10 t-test or ROC genes can be used by FSAM to achieve the greatest accuracy. Otherwise, 15 genes selected by Wilcoxon, SNR or AHP method can also be inputs to FSAM for 100% accuracy classification (see Table 6).

**Fig 11:**
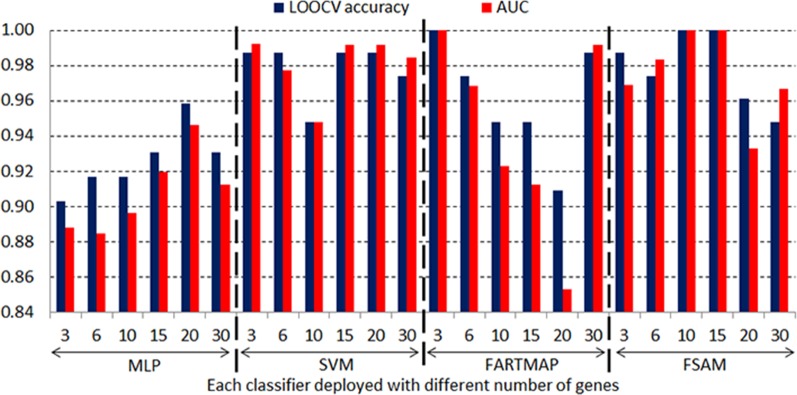
LOOCV accuracy and AUC in the DLBCL dataset with different SNR genes.

**Fig 12:**
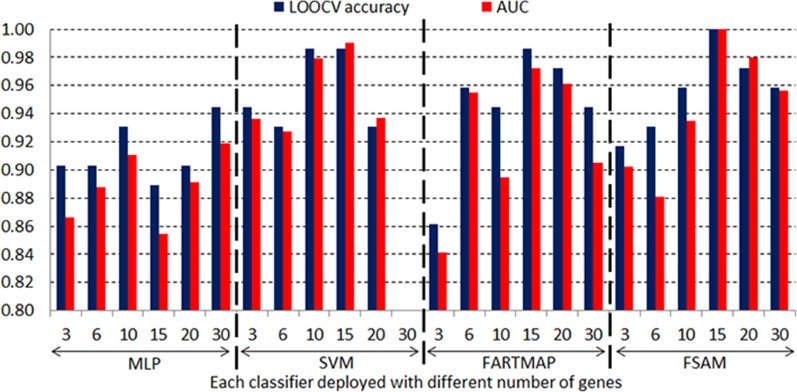
LOOCV accuracy and AUC in leukemia dataset with different SNR genes.

Fig. 11 and 12 graphically show the LOOCV accuracy and AUC obtained by four classifiers based on different number of genes driven by the SNR gene selection method in the DLBCL and leukemia datasets respectively. It is consistent with the results in Table 5 and 6 that FSAM demonstrates a steady performance through various numbers of genes. FARTMAP is very sensitive to the number of inputs and its performance hugely fluctuates when the number of genes varies. The same goes for MLP and SVM. SVM performance is rather stable in the DLBCL dataset but it diverges largely in the leukemia dataset. For example, with 15 SNR genes, SVM achieves the best with above 98% accuracy and AUC. However, it drastically drops down under 80% when 30 SNR genes are employed (see Fig. 12).

**Fig 13:**
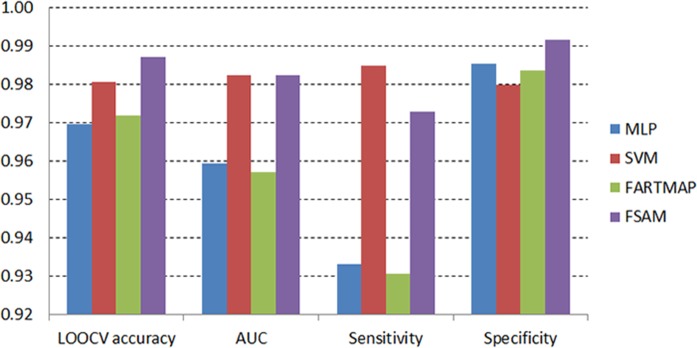
Average accuracy, AUC, sensitivity and specificity across different number of AHP genes in the DLBCL dataset.

**Fig 14:**
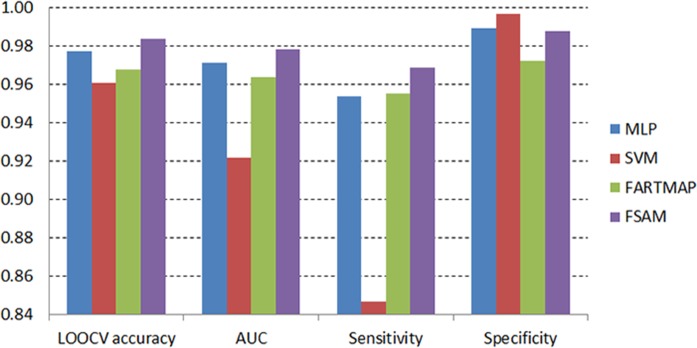
Average accuracy, AUC, sensitivity and specificity across different number of AHP genes in the leukemia dataset.

Fig. 13 and 14 on the other hand present the comparisons based on the average LOOCV accuracy, AUC, sensitivity and specificity measures of four classifiers using AHP genes in the DLBCL and leukemia datasets respectively. In line with the LOOCV accuracy measure, the FSAM performance regarding AUC also demonstrates the top ranking in both dataset.

In the DLBCL dataset, AUC of FSAM reaches the top along with that of SVM (Fig. 13). Alternatively, the AUC measure of FSAM in the leukemia dataset achieves the greatest value compared to those of MLP, SVM and FARTMAP (Fig. 14).

There is a theoretical trade-off effect between the sensitivity and specificity measures. It is showcased herein in both experiments. FSAM specificity is ranked top in the DLBCL dataset although its sensitivity is dominated by that of SVM (Fig. 13). In contrast, FSAM specificity is inferior to those of MLP and SVM in the leukemia dataset. However, its sensitivity greatly dominates those of MLP, SVM and FARTMAP (Fig. 14). Obviously, sensitivity and specificity are mutual measures as either of them increases then the other decreases and vice versa.

In general, the performance dominance of AHP-FSAM is explained by the combination between the stability of AHP-based gene subsets and the robustness of the genetic FSAM in classification. By combining outcomes of different gene ranking methods, AHP obviously generates an elite subset of genes that compile the quintessence of each individual method. On the other hand, the genetic FSAM itself deployed on various gene selection approaches also demonstrate its powerfulness and consistency in classification across different number of genes. The contradiction between the low-sample characteristic of microarray data and the rule explosion of fuzzy system is handled efficiently through employment of GA. GA reduces number of rules and thus enables FSAM to be learnt with a small number of microarray data samples. The combination proposed in this paper strengthens the classification performance by individually improving the efficiency of not only the gene selection (i.e. the modified AHP) but also that of the classifier (i.e. the genetic FSAM).

### Independent validation of the proposed method

This section presents independent validation of the proposed method by comparing it with various classifiers presented in [6, 39] using 11 datasets utilized in those papers. All datasets can be downloaded from the BRB-Array Tools Data Archive for Human Cancer Gene Expression repository at http://linus.nci.nih.gov/~brb/DataArchive_New.html. Details of each dataset are described in Table 7.

**Table 7:** Description of the datasets [6, 39].

Datasets	Descriptions
Brain Cancer	60 samples, 46 patients with classic and 14 patients with desmoplastic brain cancer
Breast Cancer 1	99 samples, patients that did (n = 45) and did not relapse (n = 54)
Breast Cancer 2	60 samples, disease-free (n = 32) or cancer recurred (n = 28)
Gastric Tumor	132 samples, 103 tumor samples and 29 normal controls
Lymphoma	58 samples. Patients that did (n = 32) and did not cured (n = 26)
Lung Cancer 1	41 samples, squamous cell lung carcinoma (21) or pulmonary carcinoid (20)
Lung Cancer 2	181 samples, 31 mesothelioma samples and 150 adenocarcinoma
Melanoma	70 samples, 45 cases of malignant melanoma patients and 25 of non-malignant patients
Myeloma	173 samples, 137 patients with bone lytic lesions,36 patients without
Pancreatic Cancer	49 samples, 24 ductal carcinoma samples and 25 normal controls
Prostate Cancer	102 samples, 50 non-tumor prostate and 52 prostate tumors

Table 8 reports results of FSAM and the competing classifiers. FSAM is implemented using a wide range number of genes [3; 6; 10; 15; 20; 30] selected by the modified AHP as in two experiments in subsection 4.2. The maximum and average LOOCV accuracy across different number of genes are presented in the first two columns of Table 8, which are denoted as FSAM (max) and FSAM (average) respectively. In the FSAM (max) column, the corresponding number of genes to the maximum accuracy is shown in brackets.

**Table 8:** Comparisons of FSAM with competing classifiers.

Datasets	FSAM (max)	FSAM (average)	MIDClass	SGC-t	SGC-W	DLDA	k-NN	SVM	RF
Melanoma	**100** (6)	**99**	98.5	97	96	97	97	97	97
Breast Cancer 1	**76** (15)	70	**76**	63	69	61	53	52	43
Brain Cancer	**87** (10)	**84**	83	80	77	65	73	60	70
Breast Cancer 2	85 (10)	81	**90**	58	50	73	67	73	67
Gastric Tumor	95 (3)	94	94	89	80	81	96	**97**	95
Lung Cancer 1	**100** (*)	**100**	98	98	95	95	98	98	98
Lung Cancer 2	**100** (15)	**99**	99	93	93	99	99	99	99
Lymphoma	**79** (10)	68	69	76	71	66	52	59	57
Myeloma	**86** (3)	**85**	84	68	67	75	78	74	79
Pancreatic Cancer	**96** (3)	**93**	78	69	90	63	61	65	55
Prostate Cancer	**96** (30)	**94**	92	89	89	78	93	93	93

^(*)^ FSAM obtains the accuracy at 100% for all experimented number of genes.

MIDClass is the notation of Microarray Interval Discriminant Classifier, which was introduced in [6] using associate rules. SGC-t and SGC-W are Single Gene Classifiers proposed in [39] based on t-test and Wilcoxon-Mann-Whitney test. DLDA, k-NN, SVM and RF denote Diagonal Discriminant Analysis [40], k-Nearest-Neighbor [41], Support Vector Machine [42] and Random Forest [43] respectively. The performances of these competing approaches are obtained from [6, 39].

The proposed FSAM outperforms the competing methods in most of the case studies (see Table 8). FSAM dominates all other methods in 9 out of 11 datasets based on the maximum column. Averaging out across different number of genes, FSAM yields larger LOOCV accuracy than other classifiers in 7 out of 11 cases. Experiments with two datasets “Breast Cancer 2” and “Gastric Tumor” show inferior performance of FSAM compared to MIDClass and SVM respectively. However, in these two case studies, FSAM actually obtains relatively great performance. For example, in the “Breast Cancer 2” dataset, its average accuracy is at 81% that is the second best among 8 competing methods. In the “Gastric Tumor” dataset, FSAM’s accuracy is at 94%, which is relatively close to the maximum 97% of the SVM classifier.

Remarkably, the accuracy of FSAM in the “Lung Cancer 1” dataset is at 100% for all experimented number of genes [3; 6; 10; 15; 20; 30]. In the “Melanoma” and “Lung Cancer 2” datasets, FSAM also achieves the maximum 100% LOOCV accuracy at 6 and 15 genes respectively.

From experimental results, we see that the average accuracy of the proposed method is obviously competent. FSAM’s performance is even more robust when it uses the optimal number of genes. The proposed approach however does not find the optimal number of genes for FSAM. This is beyond the scope of the current paper and would be addressed in a future study as mentioned in the next section.

## Concluding Remarks and Future Work

The purpose of this paper is twofold. Initially, we propose a novel method for gene selection by advancing the traditional AHP to adopt quantitative criteria that include statistics of t-test, entropy, ROC, Wilcoxon, and SNR. AHP is able to select salient expression genes through outcomes of individual ranking methods and thus it assembles the advantages of all single ones. Among the investigated gene selection methods, the AHP criterion exhibits supremacy compared to t-test, entropy, ROC, Wilcoxon and SNR methods. It is understandable as our proposed AHP is capable of deriving subsets of most informative genes as an elite collection of those raised by individual methods.

Secondly, the paper presents a new approach to cancer microarray data classification using a fuzzy system called FSAM. Via the great capability of handling noisy data with fuzzy inference, FSAM demonstrates a proficient classification technique for cancer classification through gene expression data. The traditional learning of FSAM combined with GA boost the performance of FSAM in classification. Large rule-based systems require great computational expense with a large amount of learning data. This is the reason why fuzzy systems have not attracted sufficient attention of researchers for solving effectively cancer gene expression low-sample data problems. In this paper, the application of GA to optimize the number of rules in FSAM enables the FSAM learning process to be more efficient. It also diminishes the computational cost and thus enhances the classification accuracy of FSAM.

Classification performance in this study is measured not only by the LOOCV accuracy but also by the AUC, sensitivity and specificity. The LOOCV strategy makes the experimental statistics and comparisons more meaningful as the classifiers are deployed a number of times as many as the number of data samples.

As gene selection method is important in determining the accuracy of microarray data classification, further research would concentrate on initiating different gene selection approaches by extending AHP to accommodate other ranking methods rather than just t-test, entropy, ROC, Wilcoxon and SNR. Although the performance of FSAM is successfully testified using a wide range number of genes in this study, finding the optimal number of genes for FSAM is also an interesting research that could be addressed in the future. In addition, as this study limits in experiments with binary classification, a next step to modify FSAM for multi-class problems would be worth another investigation.

## Supporting Information

S1 Table Top 30 genes selected by gene methods in the DLBCL dataset. Top 30 genes are selected by six gene selection methods: t-test, entropy, ROC, Wilcoxon, SNR and modified AHP in the DLBCL dataset.
(DOCX)

S2 Table Top 30 genes selected by gene methods in the leukemia dataset. Top 30 genes are selected by six gene selection methods: t-test, entropy, ROC, Wilcoxon, SNR and modified AHP in the leukemia dataset.
(DOCX)
