# Supplementary material for: Hierarchical Gene Selection and Genetic Fuzzy System for Cancer Microarray Data Classification
Source: PLoS One. 2015 Mar 30;10(3):e0120364. doi: 10.1371/journal.pone.0120364 (PMC4378968; doi:10.1371/journal.pone.0120364)
Supplement: S1 Table — Top 30 genes are selected by six gene selection methods: t-test, entropy, ROC, Wilcoxon, SNR and modified AHP in the DLBCL dataset. (DOCX) [file pone.0120364.s001.docx]

**Table S1. Top 30 genes selected by gene methods in the DLBCL dataset.** Top 30 genes are selected by six gene selection methods: t-test, entropy, ROC, Wilcoxon, SNR and modified AHP in the DLBCL dataset.

| No. | **T-test** | **Entropy** | **ROC** | **Wilcoxon** | **SNR** | **AHP** |
| --- | --- | --- | --- | --- | --- | --- |
| 1 | **'LDHA'** | **'P4HB'** | **'CIRBP'** | **'CIRBP'** | **'CIRBP'** | **'KPNA2'** |
| 2 | **'KPNA2'** | **'KPNA2'** | **'TRIB2'** | **'TRIB2'** | **'TRIB2'** | **'CIRBP'** |
| 3 | **'PKM2'** | **'BCL2A1'** | **'LDHA'** | **'LDHA'** | **'GPR18'** | **'P4HB'** |
| 4 | 'PSMC1' | 'HG1980-HT2023_at' | 'KPNA2' | 'KPNA2' | 'SEPP1' | 'LDHA' |
| 5 | 'SNRPB' | 'HG4074-HT4344_at' | 'PSMC1' | 'PSMC1' | 'PTPRC' | 'TRIB2' |
| 6 | 'ENO1' | 'EEF1A1_2' | 'HG1980-HT2023_at' | 'SEPP1' | 'RHOH' | 'HG1980-HT2023_at' |
| 7 | 'CIRBP' | 'LGALS3' | 'PKM2' | 'GPR18' | 'GPSM3' | 'GPR18' |
| 8 | 'ATIC' | 'LDHA' | 'CLTA' | 'HG1980-HT2023_at' | 'CCNI' | 'SEPP1' |
| 9 | 'IFI30' | 'GM2A' | 'SNRPB' | 'PTPRC' | 'TXNIP' | 'ATIC' |
| 10 | 'HG1980-HT2023_at' | 'ATIC' | 'ENO1' | 'RHOH' | 'TGFBR2' | 'SNRPB' |
| 11 | 'GM2A' | 'MT2A_2' | 'SEPP1' | 'PKM2' | 'TRA@_4' | 'GM2A' |
| 12 | 'NME1' | 'PGK1_2' | 'GPR18' | 'CLTA' | 'CD3D' | 'RHOH' |
| 13 | 'LGALS3' | 'SNRPB' | 'ATIC' | 'SNRPB' | 'HLA-A_2' | 'PSMC1' |
| 14 | 'PGAM1' | 'IFI30' | 'GM2A' | 'ENO1' | 'HNRPH1' | 'PTPRC' |
| 15 | 'CCT5' | 'IFI27' | 'PTPRC' | 'GM2A' | 'EEF1A1_2' | 'HG4074-HT4344_at' |
| 16 | 'HMGA1' | 'HG3494-HT3688_at' | 'RHOH' | 'ATIC' | 'CHI3L2' | 'PKM2' |
| 17 | 'RHOH' | 'HG417-HT417_s_at' | 'PGAM1' | 'PGAM1' | 'CLU' | 'GPSM3' |
| 18 | 'SEPP1' | 'IDH2' | 'HG2279-HT2375_at' | 'GPSM3' | 'TRBV21-1_2' | 'LGALS3' |
| 19 | 'HG417-HT417_s_at' | 'GPNMB' | 'PRDX1' | 'HG2279-HT2375_at' | 'HLA-E' | 'EEF1A1_2' |
| 20 | 'GPR18' | 'PSMC1' | 'HG4074-HT4344_at' | 'TGFBR2' | 'IL4R' | 'CCNI' |
| 21 | 'HSPD1' | 'CLTA' | 'HMGA1' | 'PRDX1' | 'NIFUN' | 'IFI30' |
| 22 | 'P4HB' | 'NME1' | 'GPSM3' | 'HG4074-HT4344_at' | 'TRBV19' | 'CLTA' |
| 23 | 'CLTA' | 'PKM2' | 'P4HB' | 'HMGA1' | 'ZFP36L2_2' | 'ENO1' |
| 24 | 'PTPRC' | 'NARS' | 'NME1' | 'P4HB' | 'CD37' | 'BCL2A1' |
| 25 | 'HG2279-HT2375_at' | 'GRN' | 'CCT5' | 'CCNI' | 'HG384-HT384_at' | 'NME1' |
| 26 | 'CCT3' | 'CSTB' | 'TGFBR2' | 'NME1' | 'SELL' | 'TGFBR2' |
| 27 | 'TRIB2' | 'TYMS' | 'HG417-HT417_s_at' | 'CCT5' | 'DDX5' | 'HG417-HT417_s_at' |
| 28 | 'GPSM3' | 'GOT2' | 'CCNI' | 'TXNIP' | 'ARPC4' | 'PGAM1' |
| 29 | 'HG4074-HT4344_at' | 'DDX21' | 'HSPD1' | 'HG417-HT417_s_at' | 'SNRPN' | 'HLA-A_2' |
| 30 | 'PRDX1' | 'CIRBP' | 'TXNIP' | 'CD3D' | 'PTK2B' | 'TXNIP' |
