# Supplementary material for: Hierarchical Gene Selection and Genetic Fuzzy System for Cancer Microarray Data Classification
Source: PLoS One. 2015 Mar 30;10(3):e0120364. doi: 10.1371/journal.pone.0120364 (PMC4378968; doi:10.1371/journal.pone.0120364)
Supplement: S2 Table — Top 30 genes are selected by six gene selection methods: t-test, entropy, ROC, Wilcoxon, SNR and modified AHP in the leukemia dataset. (DOCX) [file pone.0120364.s002.docx]

**Table S2. Top 30 genes selected by gene methods in the leukemia dataset.** Top 30 genes are selected by six gene selection methods: t-test, entropy, ROC, Wilcoxon, SNR and modified AHP in the leukemia dataset.

| No. | **T-test** | **Entropy** | **ROC** | **Wilcoxon** | **SNR** | **AHP** |
| --- | --- | --- | --- | --- | --- | --- |
| 1 | **'PSMA6'** | **'CCND3'** | **'CCND3'** | **'CCND3'** | **'FAH'** | **'CCND3'** |
| 2 | **'HG1612-HT1612_at'** | **'CD19'** | **'HG1612-HT1612_at'** | **'HG1612-HT1612_at'** | **'ATP6V0C'** | **'FAH'** |
| 3 | **'CCND3'** | **'PSMA6'** | **'FAH'** | **'FAH'** | **'PRG1'** | **'PSMA6'** |
| 4 | 'FAH' | 'RAFTLIN' | 'PSMA6' | 'PSMA6' | 'LTC4S' | 'ATP6V0C' |
| 5 | 'ATP6V0C' | 'CXCR4' | 'ATP6V0C' | 'ATP6V0C' | 'FTL' | 'HG1612-HT1612_at' |
| 6 | 'PRG1' | 'LTC4S' | 'CD81' | 'CD81' | 'TIMP2' | 'PRG1' |
| 7 | 'PSME1' | 'HG1612-HT1612_at' | 'PRG1' | 'PRG1' | 'OS9' | 'LTC4S' |
| 8 | 'CD81' | 'FTL' | 'FTL' | 'PSME1' | 'MAN2B1' | 'FTL' |
| 9 | 'CD19' | 'CD81' | 'PSME1' | 'FTL' | 'QSCN6' | 'CD19' |
| 10 | 'TIMP2' | 'MAN2B1' | 'TIMP2' | 'TIMP2' | 'TETRAN' | 'TIMP2' |
| 11 | 'MIB1' | 'XBP1' | 'MIB1' | 'MIB1' | 'AIF1' | 'OS9' |
| 12 | 'CXCR4' | 'PSMB9' | 'LTC4S' | 'PTPRCAP' | 'TALDO1' | 'MAN2B1' |
| 13 | 'PTPRCAP' | 'FAH' | 'PTPRCAP' | 'LTC4S' | 'ENO1' | 'CD81' |
| 14 | 'LTC4S' | 'PTPRCAP' | 'OS9' | 'OS9' | 'GPX1' | 'CXCR4' |
| 15 | 'AKR1B1' | 'MIB1' | 'AKR1B1' | 'AKR1B1' | 'NME4' | 'PSME1' |
| 16 | 'TCF3' | 'OS9' | 'MYB_2' | 'MYB_2' | 'ALDOA' | 'MIB1' |
| 17 | 'FTL' | 'ATP6V0C' | 'MAN2B1' | 'ADA' | 'NR1H2' | 'RAFTLIN' |
| 18 | 'STMN1' | 'PRG1' | 'ADA' | 'MAN2B1' | 'TGFB1' | 'AIF1' |
| 19 | 'MYB_2' | 'QSCN6' | 'TCF3' | 'TCF3' | 'MCL1' | 'TETRAN' |
| 20 | 'GTF2I' | 'TCF3' | 'STMN1' | 'STMN1' | 'RARA' | 'PTPRCAP' |
| 21 | 'PSMB9' | 'TETRAN' | 'PSMB9' | 'PSMB9' | 'TAGLN2' | 'QSCN6' |
| 22 | 'OS9' | 'LAPTM5' | 'CXCR4' | 'CXCR4' | 'ATP6V0B' | 'TALDO1' |
| 23 | 'MAN2B1' | 'IGHM_2' | 'NME4' | 'AIF1' | 'ITPK1' | 'NME4' |
| 24 | 'EIF3S5' | 'PSME1' | 'AIF1' | 'NME4' | 'ATP6V1F' | 'PSMB9' |
| 25 | 'IGHM_2' | 'TIMP2' | 'TALDO1' | 'TALDO1' | 'CAPNS1' | 'TCF3' |
| 26 | 'PSMB8' | 'PSMB8' | 'TETRAN' | 'GTF2I' | 'RPN2' | 'GPX1' |
| 27 | 'AIF1' | 'GTF2I' | 'GTF2I' | 'TETRAN' | 'EMP3' | 'AKR1B1' |
| 28 | 'RAFTLIN' | 'NME4' | 'ITPK1' | 'RAFTLIN' | 'ACADVL' | 'MYB_2' |
| 29 | 'SET' | 'STMN1' | 'RARA' | 'ITPK1' | 'ICAM3' | 'NR1H2' |
| 30 | 'CDK9' | 'MYB_2' | 'QSCN6' | 'CD19' | 'KCNAB2' | 'ENO1' |
